# External quality assessment for *PML‐RARα* detection in acute promyelocytic leukemia: Findings and summary

**DOI:** 10.1002/jcla.22894

**Published:** 2019-05-26

**Authors:** Qisheng Wu, Rui Zhang, Yu Fu, Jiawei Zhang, Kun Chen, Jinming Li

**Affiliations:** ^1^ National Center for Clinical Laboratories, Beijing Hospital National Center of Gerontology Beijing China; ^2^ Graduate School, Peking Union Medical College Chinese Academy of Medical Sciences Beijing China; ^3^ Beijing Engineering Research Center of Laboratory Medicine Beijing Hospital Beijing China

**Keywords:** acute promyelocytic leukemia, external quality assessment, *PML‐RARα*, real‐time quantitative reverse transcription PCR

## Abstract

**Background:**

The confirmation of clinical diagnosis, molecular remission, and sequential minimal residual disease monitoring required *PML‐RARα* detection in acute promyelocytic leukemia (APL). The current status of *PML‐RARα* detection in various laboratories remains unknown.

**Methods:**

In 2018, external quality assessment (EQA) for *PML‐RARα* detection was carried out in China. Three EQA sample panels for *PML‐RARα* isoform L/S/V were prepared by different mock leukocyte samples. The performances of *PML‐RARα* detection, including admission screening, and qualitative and quantitative detection by real‐time quantitative reverse transcription PCR (RT‐qPCR), were assessed based on APL simulated clinical case.

**Results:**

The mock leukocyte samples met the requirements of a clinically qualified sample for *PML‐RARα* EQA panel. Among the laboratories, 13/50 (26.0%) were “competent,” 21/50 (42%) classified as “acceptable,” and 16/50 (32.0%) classified as “improvable.” One (1/50, 2.0%) laboratory reported one screening mistake. Twenty‐six (26/50, 52.0%) laboratories reported 29 false‐positive and 19 false‐negative results. Twenty‐three (23/50, 46.0%) laboratories reported 42 quantitative incorrect results.

**Conclusion:**

Significant differences were not found in *PML‐RARα* detection performance among laboratories that used different extraction methods. The performances of qualitative and quantitative RT‐qPCR detection were worse accurate for *PML‐RARα* isoform V. Quantitative variation was higher for low‐level samples. Further continuous external assessment and education are needed in the management of *PML‐RARα* detection.

## 1 INTRODUCTION

Acute promyelocytic leukemia (APL) is a distinct subtype of acute myeloid leukemia (AML) with characteristic biological and clinical features,^1^ comprising approximately 10% of de novo AML cases in younger adults.^2^ APL is present of a specific t(15;17) chromosomal translocation in the leukemic blast, which involves the promyelocyte (*PML*) gene on chromosome 15 to the retinoic acid receptor‐alpha (*RARα*) gene on chromosome 17.^3^ According to different breakpoints in *PML* and *RARα*, there are three isoforms of *PML‐RARα* fusion gene (FG): long (L, 55%), variant (V, 5%), and short (S, 45%).^4^

*PML‐RARα* FG is present in almost all APL cases and is a biomarker for APL diagnosis, disease burden, minimal residual disease (MRD) monitoring, and molecular remission.^5^, ^6^, ^7^ Detection methods for t(15;17) or *PML‐RARα* FG include conventional chromosome analysis, fluorescence in situ hybridization, and polymerase chain reaction (PCR). Compared with common reverse transcription PCR (RT‐PCR), real‐time quantitative reverse transcription PCR (RT‐qPCR) for *PML‐RARα* has higher precision and reliability, and is routinely used, especially in molecular hematology laboratories.^8^

Clinical detection of the *PML‐RARα* fusion gene is important in APL development. APL can be diagnosed in patients with abnormal hematopoiesis and characteristic cytogenetic abnormalities with t(15;17), regardless of the percentage of marrow blasts.^9^ *PML‐RARα* FG transcript level can reflect the abnormal leukemia blasts load, quantitatively document disease burden, and confirm molecular remission.^10^ The goal of consolidation therapy for APL is a durable molecular remission, defined as undetectable *PML‐RARα* FG.^7^, ^11^ Rigorous sequential MRD monitoring by RT‐qPCR coupled with pre‐emptive therapy can help reduce clinical relapse rates in APL patients.^5^, ^8^

External quality assessment (EQA) programs of common RT‐PCR for *PML‐RARα* FG test were first performed nearly 20 years ago.^12^, ^13^ These programs used RNA, cDNA, or plasmid as EQA samples, and examined the heterogeneous sensitivities of *PML‐RARα* FG RT‐PCR detection. In 2003, the *Europe Against Cancer* (EAC) program established RT‐qPCR standardization and quality control analysis for the *PML‐RARα* FG transcript and recommended the ratio of FG copy number to control genes (CG) copy number (FG_CN_/CG_CN_) as the *PML‐RARα* FG transcript level.^14^, ^15^ The MRD value is a ratio between the FG transcript level in follow‐up ((FG_CN_/CG_CN_)_FUP_) and diagnostic samples ((FG_CN_/CG_CN_)_DX_).^14^, ^15^ These studies promoted the improvement of the PCR detection sensitivity and accuracy for *PML‐RARα* FG, especially the EAC‐sanctioned RT‐qPCR. However, there existed some limitations. For some detection defects, total RNA, cDNA, recombinant plasmid, and NB4 cells were not suitable as EQA samples.^16^, ^17^ Little is known about the evaluation of *PML‐RARα* isoform V detection. These EQA programs only assessed the accuracy of the RT‐PCR or RT‐qPCR methodology, but did not analyze MRD monitoring results for *PML‐RARα* based on APL clinical information.^5^, ^6^, ^7^ The EQA scoring criteria for *BCR‐ABL1* are unsuitable for *PML‐RARα*, because only the accuracy of quantitative RT‐qPCR detection was analyzed, with no admission screening and qualitative test.^18^

We made MS2 armored RNAs for *PML‐RARα* FG transcript, CG transcript, and 23s rRNA. Armored RNAs are stable, nuclease‐resistant, and precisely quantifiably synthetic RNAs. They were already used as *BCR‐ABL1* and control gene standards.^19^, ^20^ The EQA panel of *PML‐RARα* isoform L/V/S with simulated APL clinical information was designed. We prepared mock leukocyte samples as EQA samples by mixing different amounts of the aforementioned armored RNAs, which can simulate total RNA yields extracted from BM by adding a large amount of 23s rRNA armored RNA. The *PML‐RARα* detection was assessed, including RNA extraction, admission screening, and qualitative and quantitative RT‐qPCR test.

## 2 MATERIALS AND METHODS

### 2.1 Design of APL simulated case

According to the *NCCN Clinical Practice Guidelines in Oncology Acute Myeloid Leukemia (version 3.2017)* and *Management of acute promyelocytic leukemia: recommendations from an expert panel on behalf of the European LeukemiaNet*, we designed APL simulated clinical case for isoforms L/S/V^6^, ^7^ (see Appendix S1).

### 2.2 Preparation and evaluation of mock leukocyte samples for EQA panel

Total RNA extracted from BM was divided into three components, including *PML‐RARα* FG transcript RNA, CG transcript RNA, and other non‐target RNA. We used MS2 virus‐like particle packaging technology^21^ to make mock leukocytes samples, which consisted armored RNAs of *PML‐RARα* FG L/V/S (AR‐FG L/V/S), chimeric CGs (AR‐CG), and 23s rRNA (AR‐23s). Firstly, The recombinant plasmids, pACYC‐MS2‐*PML‐RARα* L/V/S, pACYC‐MS2‐CGs, and pACYC‐MS2‐23s rRNA, were constructed separately. Then, five armored RNAs were expressed and purified as previously described.^22^, ^23^ The armored RNAs were identified by transmission electron microscopy, enzymatic digestion test, sodium dodecyl sulfate‐polyacrylamide gel electrophoresis, and RT‐PCR.

We constructed *PML‐RARα* FG EQA panel which consisted of limited positive and negative samples with different FG_CN_/CG_CN_ ratio and MRD value (Table 1). The positive mock leukocyte samples were obtained by mixing AR‐FG L/V/S and AR‐CG at different concentrations after adding 30 μL AR‐23s. Negative mock leukocyte samples were prepared from specified concentrations of AR‐CG after adding 30 μL AR‐23s. All mock leukocyte samples were freeze‐dried and stored at −20°C.

**Table 1:** Composition of the *PML‐RARα* FG EQA panel and RT‐qPCR results

EQA panel	Sample No.	Isoform classification	FG_CN_/CG_CN_ ratio	MRD value	Log reduction	No. of correct/Total no. tested (%)
A	A1711	L	10.00%	1	0	25/25 (100)
	A1712	L	2.00%	0.2	0.6990	22/25 (88)
	A1713	Negative	0	Negative	Negative	22/25 (88)
	A1714	Negative	0	Negative	Negative	24/25 (96)
	A1715	L	0.02%	0.002	2.6990	19/25 (76)
B	B1721	S	10.00%	1	0	25/25 (100)
	B1722	S	2.00%	0.2	0.6990	17/25 (68)
	B1723	Negative	0	Negative	Negative	20/25 (80)
	B1724	Negative	0	Negative	Negative	20/25 (80)
	B1725	S	0.02%	0.002	2.6990	22/25 (88)
C	C1731	V	130.00%	1	0	49/50 (98)
	C1732	Negative	0	Negative	Negative	42/50 (84)
	C1733	V	0.02%	0.00015	3.8239	35/50 (70)
	C1734	Negative	0	Negative	Negative	43/50 (86)
	C1735	V	0.21%	0.0015	2.8240	36/50 (72)
	C1736	V	10.00%	0.077	1.1135	36/50 (72)

FG_CN_/CG_CN_ ratio, fusion gene copy number/control gene copy number.

The EQA panel was evaluated using a routine detection process. Total RNA was extracted by TRIzol reagent and spin column, quantified using NanoDrop 2000c (Thermo Fisher). Using the one‐step or two‐step RT‐qPCR method, qualitative and quantitative detection of *PML‐RARα* FG and CG was performed by the manufacturer's instructions on ABI 7500 Instrument (Applied Biosystems).

### 2.3 Organization of the EQA

Before sample processing, the EQA samples should be centrifuged at 12 000 r/min for 1 min and did not need the reconstitution and the lysis of red blood cells. Total RNA extraction was performed by using routine operating procedure of individual laboratory. Participating laboratories first performed screening tests for the admission sample (A1711, B1721, and C1731) based on APL simulated clinical case; then, RT‐qPCR was carried out, and the FG_CN_/CG_CN_ ratio and MRD value were calculated. EQA panel A or B set was randomly assigned to the participants beside EQA panel C delivery to all laboratories. Each participant was asked to report the results on the data sheet within 2 weeks.

### 2.4 Laboratory performance scoring

Accurate detection of the *PML‐RARα* FG was prerequisite for APL diagnosis and MRD monitoring.^6^, ^7^, ^9^ Any result distinct from the established value was considered as “incorrect result” which will affect evaluation of treatment effect for APL MRD. Any error in RT‐qPCR is multiplicative, rather than additive, data distributions from RT‐qPCR–based EQA testing program produce a lognormal distribution, that is an asymmetric distribution of results with a strong positive skew.^24^ The log reduction was calculated by using the admission sample in each EQA panel as the baseline. The reduction in *PML‐RARα* levels from this baseline value was then calculated for each correct qualitative positive EQA sample and reported as a log reduction.^18^, ^25^, ^26^ The log reduction was analyzed using a robust statistical Z‐score,^27^ the score ≥3 as “incorrect result”.

The EQA scores based on qualitative and quantitative results were classified as “competent” (100% satisfied results), “acceptable” (<2 incorrect results), or “improvable” (more than 2 incorrect results).

### 2.5 Statistical analyses

All data were analyzed using SPSS version 16.0. *PML‐RARα* detection sensitivity, specificity, accuracy, and variation distribution between different samples or groups were compared using *t* test or one‐way ANOVA or Fisher chi‐square test. *P* values < 0.05 were considered statistically significant.

## 3 RESULTS

### 3.1 Quality assessment of armored RNAs

**Figure 1:**
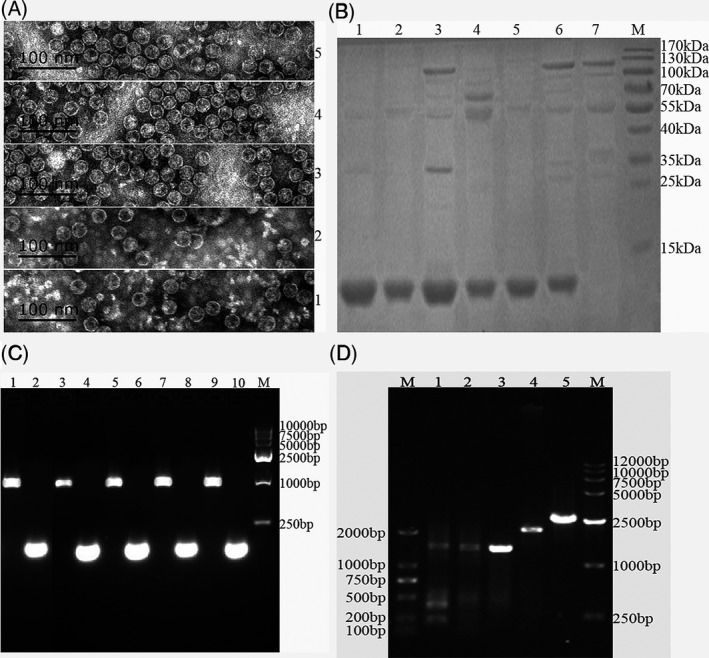
Evaluation of armored RNAs. A, Identification of five armored RNAs by transmission electron microscopy. The diameter of armored RNAs was approximately 30 nm. Number 1, 23s rRNA armored RNA (AR‐23s); Number 2, chimeric CGs armored RNA (AR‐CG); Number 3, *PML‐RARα* FG L armored RNA (AR‐FG L); Number 4, *PML‐RARα* FG S armored RNA (AR‐FG S); and Number 5, *PML‐RARα* FG V armored RNA (AR‐FG V). B, After purification by gel exclusion chromatography, freshly prepared armored RNAs were loaded onto an SDS‐polyacrylamide gel and subjected to electrophoresis in tricine buffer. Proteins were visualized by staining the gel with Coomassie brilliant blue. Lane M, PageRuler Prestained Protein Ladder; Lane 1, AR‐23s; Lane 2, AR‐CG; Lane 3, AR‐FG L; Lane 4, AR‐FG S; Lane 5, AR‐FG V; Lane 6, negative control (blank); and Lane 7, positive control (MS2). C, Identification of five armored RNA by agarose gel electrophoresis after enzymatic digestion test. Freshly prepared armored RNAs were incubated with RNase A and DNase I at 37°C for 1 h and subsequently analyzed on a 1% agarose gel, producing bands between 1 kb and 2 kb. Lane M, molecular weight marker; Lane 1, AR‐23s without incubation with RNase A and DNase I; Lane 2, AR‐23s incubated with RNase A and DNase I; Lane 3, AR‐CG without incubation with RNase A and DNase I; Lane 4, AR‐CG incubated with RNase A and DNase I; Lane 5, AR‐FG L without incubation with RNase A and DNase I; Lane 6, AR‐FG L incubated with RNase A and DNase I; Lane 7, AR‐FG S without incubation with RNase A and DNase I; Lane 8, AR‐FG S incubated with RNase A and DNase I; Lane 9, AR‐FG V without incubation with RNase A and DNase I; and Lane 10, AR‐FG V incubated with RNase A and DNase I. D, Ethidium bromide–stained 1% agarose gel of RT‐PCR amplification products of RNA extracted from armored RNAs. Lane M, two kinds of molecular weight marker; Lane 1, RT‐PCR products of RNA extracted from AR‐FG L; Lane 2, RT‐PCR products of RNA extracted from AR‐FG S; Lane 3, RT‐PCR products of RNA extracted from AR‐FG V; Lane 4, RT‐PCR products of RNA extracted from AR‐CG; and Lane 5, RT‐PCR products of RNA extracted from AR‐23s

Armored RNAs were constructed and expressed successfully by validation of sequencing and a series of experiments (Figure 1), and by TEM to detect the diameters of the armored RNAs of MS2 VLPs (about 30 nm; Figure 1A) and SDS‐PAGE to clear molecular weight of proteins (about 14 KD; Figure 1B). Digesting with RNase A and DNase I for 1 hour at 37°C, only one single band between 1 kb and 2 kb was visible using 1% agarose gel electrophoresis (Figure 1C). RT‐PCR was performed respectively to confirm encapsulation of the five target sequences (Figure 1D), followed by sequencing. To verify their stability and availability of the armored RNAs for the EQA study before panel distribution, stability analyses were performed and approved that armored RNAs were stable for at least 2 weeks at 37°C, room temperature, 4°C, and −20°C (data not shown).

### 3.2 Evaluation of mock leukocyte samples

Total RNA yields extracted by TRIzol reagent ranged from 15.2 to 24.5 μg/sample and spin column from 9.3 to 14.1 μg/sample, respectively. The quantitative validation results of the positive samples are slightly different between one‐step and two‐step method which is in agreement with expected values (See Appendix S2).

### 3.3 Panel distribution and response

Fifty laboratories submitted their detection results and experimental data. TRIzol reagent was widely used by 46/50 (92%) participants, the spin column method was used by 3/50 (6%) laboratories, and only one laboratory used the magnetic bead method. The 37/50 (74%) laboratories used *PML‐RARa* fusion gene RT‐qPCR kit (YUANQI BIO Co., Ltd. Shanghai, China) for one‐step method, and 12/50 (24%) laboratories used two‐step in‐house RT‐qPCR. One laboratory (2%) used *PML‐RARa* fusion gene RT‐qPCR kit (SYBio Co., Ltd. Shanghai, China) for two‐step method (Table 2).

**Table 2:** Qualitative and quantitative performance of different assays for each EQA panel

EQA panel	Assay	YUANQI			SY		In‐house	
		Qualitative		Quantitative	Qualitative	Quantitative	Qualitative	Quantitative
	No. of data sets	37			1		12	
A	A1711	19/19 (100)	19/19 (100)		NT	NT	6/6 (100)	6/6 (100)
	A1712	19/19 (100)	16/19 (84.2)		NT	NT	6/6 (100)	6/6 (100)
	A1713	16/19 (84.2)	/		NT	NT	6/6 (100)	/
	A1714	18/19 (94.7)	/		NT	NT	6/6 (100)	/
	A1715	18/19 (94.7)	13/18 (72.2)		NT	NT	6/6 (100)	6/6 (100)
B	B1721	18/18 (100)	18/18 (100)		1/1 (100)	1/1 (100)	6/6 (100)	6/6 (100)
	B1722	17/18 (94.4)	11/17 (64.7)		1/1 (100)	1/1 (100)	6/6 (100)	5/6 (83.3)
	B1723	14/18 (77.8)	/		1/1 (100)	/	5/6 (83.3)	/
	B1724	14/18 (77.8)	/		1/1 (100)	/	5/6 (83.3)	/
	B1725	18/18 (100)	17/18 (94.4)		1/1 (100)	0/1 (0)	5/6 (83.3)	5/5 (100)
C	C1731	36/37 (97.3)	37/37 (100)		1/1 (100)	1/1 (100)	12/12 (100)	12/12 (100)
	C1732	33/37 (89.2)	/		1/1 (100)	/	8/12 (66.7)	/
	C1733	30/37 (81.1)	25/29 (86.2)		0/1 (0)	0	12/12 (100)	11/12 (91.7)
	C1734	33/37 (89.2)	/		1/1 (100)	/	9/12 (75.0)	/
	C1735	32/37 (84.5)	26/32 (81.3)		0/1 (0)	0	12/12 (100)	10/12 (83.3)
	C1736	36/37 (97.3)	25/36 (69.4)		1/1 (100)	1/1 (100)	12/12 (100)	10/12 (83.3)
Total		371/407 (91.2)	207/243 (85.2)		9/11 (81.8)	4/5 (80.0)	122/132 (92.4)	78/83 (94.0)
Sensitivity	94.6% (331/350)	93.8% (243/259)			71.4% (5/7)		98.8% (83/84)	
Specificity	85.5% (171/200)	86.5% (128/148)			100% (4/4)		81.3% (39/48)	
Accuracy	91.3% (502/550)	91.2% (371/407)			81.8% (9/11)		92.4% (122/132)	

In‐house, in‐house‐developed RT‐qPCR assay; NT, not tested; SY, *PML‐RARa* fusion gene RT‐qPCR kit (Shanghai SYBio Co., Ltd.); YUANQI, *PML‐RARa* fusion gene RT‐qPCR kit (Shanghai YUANQI BIO Co., Ltd.).

### 3.4 Performance of laboratories

**Figure 2:**
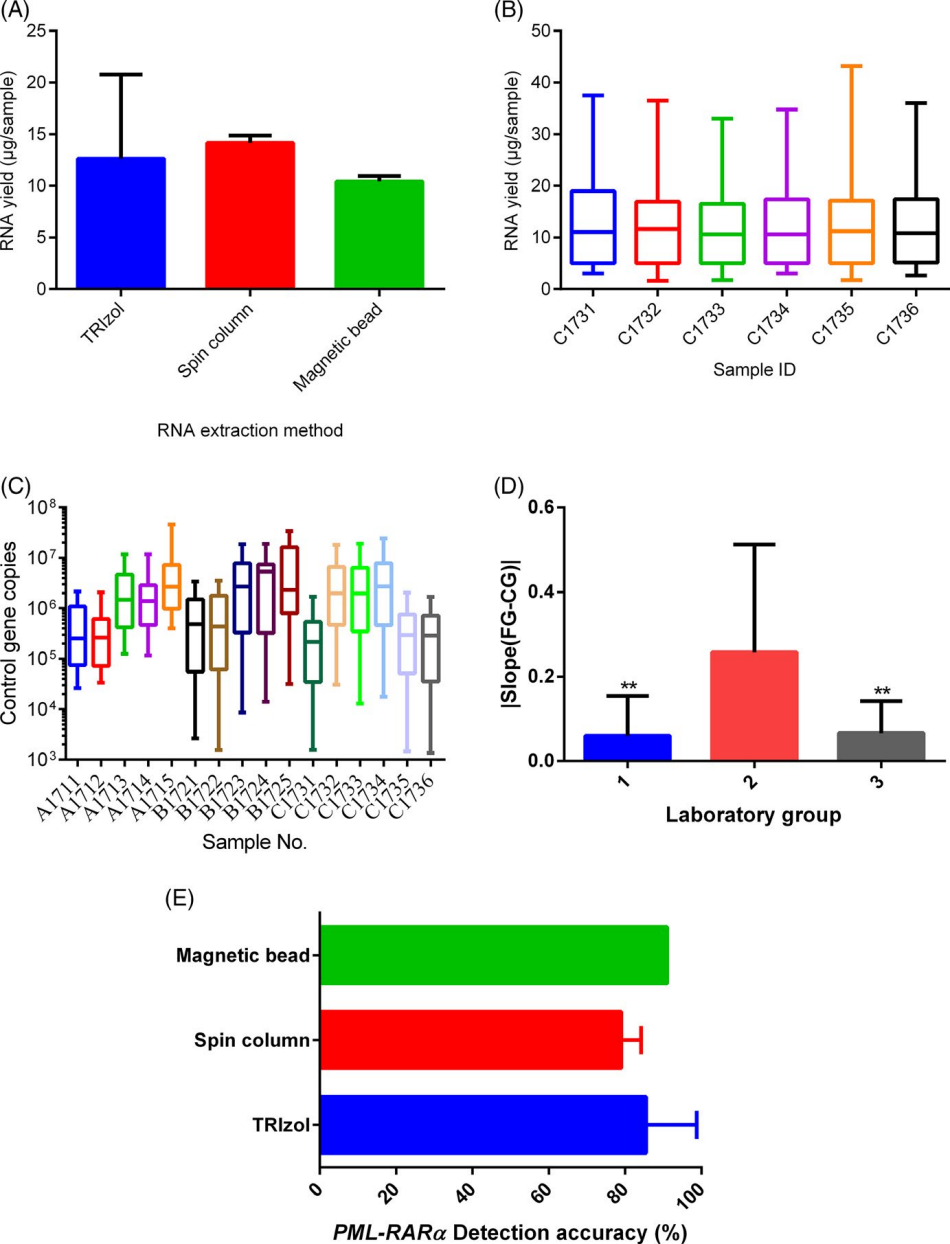
Detection performance of mock leukocyte samples for EQA panels. A, RNA yields extracted by different methods. Error bars represent standard deviation. B, RNA yields extracted by TRIzol between EQA samples. C, Control gene copy number of each mock leukocyte sample. D, Differences in the slope of RT‐qPCR standard curve between different laboratory groups. 1, correct detection group; 2, quantitative incorrect group; 3, only qualitative incorrect group. Error bars represent standard deviation. **P < 0.001 vs 2. E, Effect of different RNA extraction methods for *PML‐RARα* detection accuracy

The mock leukocyte samples had good adaptability to various RNA extraction methods. We did not find significant differences in RNA extraction performance among laboratories that used different extraction methods (*P* = 0.79; Figure 2A). RNA yields extracted by TRIzol reagent between EQA samples in panel C were consistent (*P* = 0.99; Figure 2B). All 50 laboratories used *ABL1* as the control gene. Excluding 6 results from one laboratory, other laboratories had control gene *ABL1* CN >10^4^ and the median of CG CN ranged from 1.14 × 10^4^ to 4.57 × 10^7^ (Figure 2C). The different RNA extraction methods had no effect on *PML‐RARa* detection accuracy and no significant difference (*P* = 0.40; Figure 2E).

Among the laboratories, 13/50 (26.0%) laboratories were “competent,” 21/50 (42%) classified as “acceptable,” and 16/50 (32.0%) classified as “improvable.” The performances of the different RT‐qPCR assays used for the qualitative and quantitative tests indicated overall accuracy, sensitivity, and specificity were 91.1%, 94.0%, and 86.0%, respectively; the accuracy of in‐house methods was better than commercial kits, and EQA panel C for isoform V detection was worse than that of EQA panels A and B (Tables 1 and 3).

**Table 3:** Qualitative incorrect results of different reagents and EQA panels

Incorrect results	Reagent type	No. of qualitative incorrect results for EQA panel (No. of laboratory)			
		A	B	C	Total
False‐negative	Isoform classified	1 (1)	1 (1)	4 (3)	6 (4)
	Not classified	0 (0)	1 (1)	11 (5)	12 (6)
False‐positive	Isoform classified	4 (3)	8 (4)	11 (11)	23 (15)
	Not classified	0 (0)	2 (1)	5 (4)	7 (4)
	Total	5 (4)	12 (7)	31 (21)	48 (26)

Concerning admission screening test, 49 (49/50, 98.0%) participating laboratories were excellently proficient. One laboratory made *PML‐RARa* FG isoform V identification mistake. Forty laboratories (40/50, 80.0%) identified isoform L/S/V, while the remaining 10 (10/50, 20.0%) did not.

Of 550 qualitative results received, 48 (8.7%) were incorrect, including 18 false‐negative (FN) results and 30 false‐positive (FP) results. In 18 FN results, case set C accounted for 15 incorrect results, with the remaining 2 ones from B, and one from A. Of 30 FP results, case sets A/B/C occupied 4, 10, and 16 incorrect results, respectively (Table 3). Six laboratories that did not apply isoform‐classified reagents reported 19 incorrect qualitative results, 12 FN results, and 7 FP results; 16 isoform‐classified laboratories had 29 incorrect results, 6 FN, and 23 FP (Table 3).

In quantitative RT‐qPCR test, 42 incorrect results were reported by 23 participating laboratories. The mean, median, standard deviation (SD), and coefficient of variation (CV) of log reduction for *PML‐RARα* quantitative results are summarized (Table 4). The CV of case set C was greater than the value of case sets A and B. The CV value increased at a higher *PML‐RARα* level in each sample set (Table 4). Case sets A/B/C contributed 8, 8, and 26 incorrect results, separately. The quantitative accuracy of in‐house methods was higher than that of commercial kits (*P* = 0.036; Table 2). The slope and *R* ^2^ value of the standard curve for quantitative RT‐qPCR were analyzed in participating laboratories. The range was from −2.19 to −4.15 for the slope and 0.96 to 1.00 for the *R* ^2^ value. According to RT‐qPCR quantitative results, we divided the participating laboratories into 3 groups, including correct detection group, quantitative incorrect group, and only qualitative incorrect group. Using the difference in slope as an index, the inconsistencies in amplification efficiency of *PML‐RARα* FG and CG in the quantitative incorrect group were statistically significantly greater than those in the other two groups (Figure 2D).

**Table 4:** Summary of quantitative test variation at different EQA panels

Log reduction	EQA panel A		EQA panel B		EQA panel C		
	A1712	A1715	B1722	B1725	C1733	C1735	C1736
Mean	0.6376	2.6109	0.7012	2.7032	3.5326	2.5189	1.2524
SD	0.0629	0.1577	0.0648	0.2184	0.4545	0.3255	0.1813
CV (%)	9.87	6.04	9.24	8.08	12.87	12.92	14.48
Median	0.6675	2.6244	0.7007	2.7241	3.5066	2.5149	1.2182
Minimum	0.1260	2.1543	0.2273	2.2450	2.2040	1.6345	‐0.6487
Maximum	1.1837	3.7677	1.1222	3.4919	4.2936	3.8516	2.2852
Range	1.0577	1.6134	0.8949	1.2469	2.0896	2.2171	2.9339
No.	25	24	24	24	42	44	49

CV, coefficient of variation; SD, standard deviation.

## 4 DISCUSSION

We successfully designed mock leukocyte samples as the EQA panel for qualitative and quantitative RT‐qPCR detection performance. Thirty‐seven of the laboratories reported incorrect qualitative or quantitative results of *PML‐RARα* detection. The detection performance of the laboratories using in‐house methods for *PML‐RARα* was significantly better than those using commercial reagents. Among three sample sets, the detecting ability to rare isoform V was worse than L or S. In the same sample set, the detection accuracy of *PML‐RARα* low‐level samples was lower than the high‐level samples which prompt these participants needed to improve RT‐qPCR test reliability.

The mock leukocyte samples met the requirements of a clinically qualified sample for *PML‐RARα* EQA panel. By mixing different armored RNAs of *PML‐RARα* FG, CG, and 23s rRNA, we simulated the composition and RNA yield of total RNA of BM‐nucleated leukocytes to prepare mock leukocyte samples. The qualified samples were judged by copy number (CN) of control gene, *ABL1* is >10^4^, or location of Ct value is in the range of 21.9–29.3.^14^, ^15^, ^30^ The copy number of control gene in each EQA sample tested by laboratories complied with the above requirements. RNA yields extracted by TRIzol reagent between EQA samples were consistent with the range of RNA yields/sample reported.^28^ The RNA yields did not seem to affect *PML‐RARα* FG RT‐qPCR detection, because laboratories achieved accurate detection results (Figure 2E, Table 2). This conclusion was consistent with previous findings.^26^

The degree of agreement with the established value reached 91.3% (502/550) in *PML‐RARα* qualitative RT‐qPCR detection for all EQA samples. This qualitative accuracy rate was slightly higher than previously reported.^12^ Except for accidental specimen loading errors, laboratory aerosol and instrument contamination may be the main cause of false‐positive results, especially for ultra‐low *PML‐RARα* copies. By analyzing the reported RT‐qPCR false‐positive results, we found that laboratories using in‐house method had more false‐positive results than those using commercial reagents. The in‐house method needs to open the lid between cDNA synthesis step and PCR amplification step, and there is an increased chance of residual contamination. In addition, the intensive distribution of EQA‐positive samples, larger load volumes, and repeated detection of specific samples may have more opportunities for cDNA synthesis and PCR contamination.

We found that commercial reagents had lower sensitivity than in‐house method. Five laboratories using commercial unclassified reagents reported 11 false‐negative results for medium‐level and low‐level isoform V samples. This may be due to the low detection sensitivity of the *PML‐RARα* unclassified reagents for rare isoform V, especially low‐level sample. These laboratories were obliged to improve program documentation to accommodate *PML‐RARα* rare isoform V detection. Only one laboratory reported *ABL1* CG <10^4^ copies, and the FN results were due to incorrect preservation of RNA of the laboratory leading to RNA degradation.

There was a great deal of *PML‐RARα* FG quantitative variation between not only reagents but also case sets (Table 4). We observed that commercial reagents reported more quantitative improper results than in‐house method, especially for *PML‐RARα* isoform V (Table 2). The possible reasons for that are as follows. The inconsistency of the RT‐qPCR detection efficiency between each sample and every EQA case sets accompanies a evident divergence. The variation of RT‐qPCR quantitative detection for commercial reagents (YUANQI) was greater than in‐house methods, especially the high *PML‐RARα* level (Table 4). The quantitative variation had a lot to do with the intrinsic procedure, for example, the determination of standard curve. The higher difference in slope of *PML‐RARα* FG and CG in the quantitative incorrect group was in charge of quantitative wrong (Figure 2D). In addition, unequal amplification efficiency between the plasmid calibration standard and the RNA template will bring about an potential augment in quantitative detecting inaccuracy^29^; thus, standard curve should satisfy both slope range (from‐3.2 to −3.6) and *R* ^2^ > 0.980 like BCR‐ABL1.^30^ Laboratories should optimize and validate the RT‐qPCR procedures to achieve consistent quantitative detection capacity of different isoforms.

Mock leukocyte samples successfully can be used to assess *PML‐RARα* detection. Significant differences were not found in *PML‐RARα* detection performance among laboratories that used different extraction methods. The performances of qualitative and quantitative RT‐qPCR tests were worse in the *PML‐RARα* detection process, especially for *PML‐RARα* FG rare isoform V. Quantitative variation was higher for fusion gene low‐level samples. To improve *PML‐RARα* FG detection, laboratories should conduct internal quality control and anti‐contamination, optimize RT‐qPCR methodology, and regularly maintain and calibrate PCR instrument to ensure the accuracy of qualitative and quantitative detection. Our study highlights the need for further continuous external assessments and education in the management of APL *PML‐RARα* detection process.

## CONFLICT OF INTEREST

The authors declare that they have no conflict of interest.

## AUTHORS’ CONTRIBUTIONS

Qisheng Wu designed the research study, performed the research, analyzed the data, and wrote the manuscript; Jinming Li reviewed and critically revised the manuscript, and approved the submitted and final versions; Rui Zhang reviewed and critically revised the manuscript; Yu Fu, Jiawei Zhang, and Kun Chen performed the research.

## References

[1] Tallman MS , Kwaan HC . Reassessing the hemostatic disorder associated with acute promyelocytic leukemia. Blood. 1992;79:543‐553. 1732003

[2] Park Jh , Qiao B , Panageas Ks , et al. Early death rate in acute promyelocytic leukemia remains high despite all‐trans retinoic acid. Blood. 2011;118:1248‐1254. 21653939 10.1182/blood-2011-04-346437 PMC3790946

[3] Grignani F , Ferrucci PF , Testa U , et al. The acute promyelocytic leukemia‐specific PML‐RARα fusion protein inhibits differentiation and promotes survival of myeloid precursor cells. Cell. 1993;74:423‐4314. 8394219 10.1016/0092-8674(93)80044-f

[4] Borrow J , Goddard AD , Sheer D , Solomon E . Molecular analysis of acute promyelocytic leukemia breakpoint cluster region on chromosome 17. Science. 1990;249:1577‐1580. 2218500 10.1126/science.2218500

[5] Grimwade D , Jovanovic JV , Hills RK , et al. Prospective minim‐al residual disease monitoring to predict relapse of acute promyelocytic leukemia and to direct pre‐emptive arsenic trioxide therapy. J Clin Oncol. 2009;27:3650‐3658. 19506161 10.1200/JCO.2008.20.1533

[6] Jun M . Chinese Society of Hematology C M. Chinese guidelines for diagnosis and treatment of acute promyelocytic leukemia. Zhonghua Xue Ye Xue Za Zhi. 2014;35:475‐477. 24857227 10.3760/cma.j.issn.0253-2727.2014.05.024

[7] Kim HL , Puymon MR , Qin M , Guru K , Mohler JL . NCCN clinical practice guidelines in oncology™ Acute Myeloid Leukemia. 2017:Version 1.

[8] Lange AP , Lima AS , Lucena‐Araujo AR , et al. The experience of the International Consortium on Acute Promyelocytic Leukemia in monitoring minimal residual disease in acute promyelocytic leukaemia. Br J Haematol. 2016;144:90‐92. 10.1111/bjh.14490 28025822

[9] Arber DA , Orazi A , Hasserjian R , et al. The 2016 revision to the World Health Organization (WHO) classification of myeloid neoplasms and acute leukemia. Blood. 2016;127:2391‐2405. 27069254 10.1182/blood-2016-03-643544

[10] Grimwade D , Coco FL . Acute promyelocytic leukemia: a model for the role of molecular diagnosis and residual disease monitoring in directing treatment approach in acute myeloid leukemia. Leukemia. 2002;16:1959‐1973. 12357347 10.1038/sj.leu.2402721

[11] Cheson BD , Bennett JM , Kopecky KJ , et al. Revised recommendations of the international working group for diagnosis, standardization of response criteria, treatment outcomes, and reporting standards for therapeutic trials in acute myeloid leukemia. J Clin Oncol. 2003;21:4642‐4649. 14673054 10.1200/JCO.2003.04.036

[12] Bolufer P , Barragán E , Sánz Ma , et al. Preliminary experience in external quality control of RT‐PCR PML‐RARα detection in promyelocytic leukemia. Leukemia. 1998;12:2024‐2028. 9844933 10.1038/sj.leu.2401225

[13] Bolufer P , Coco FL , Grimwade D , et al. Variability in the levels of PML‐RAR alpha fusion transcripts detected by the laboratories participating in an external quality control program using several reverse transcription polymerase chain reaction protocols. Haematologica. 2001;86:570‐576. 11418365

[14] Gabert J , Beillard E , van der Velden V , et al. Standardization and quality control studies of ‘real‐time’ quantitative reverse transcriptase polymerase chain reaction of fusion gene transcripts for residual disease detection in leukemia–a Europe Against Cancer program. Leukemia. 2003;17:2318‐2357. 14562125 10.1038/sj.leu.2403135

[15] Beillard E , Pallisgaard N , van der Velden V , et al. Evaluation of candidate control genes for diagnosis and residual disease detection in leukemic patients using ‘real‐time’ quantitative reverse‐transcriptase polymerase chain reaction (RQ‐PCR)–a Europe against cancer program. Leukemia. 2003;17:2474‐2486. 14562124 10.1038/sj.leu.2403136

[16] Drexler HG , Matsuo Y . Guidelines for the characterization and publication of human malignant hematopoietic cell lines. Leukemia. 1999;13:835‐842. 10360369 10.1038/sj.leu.2401428

[17] Filatov L , Golubovskaya V , Hurt JC , Byrd LL , Phillips JM , Kaufmann WK . Chromosomal instability is correlated with telomere erosion and inactivation of G2 checkpoint function in human fibroblasts expressing human papillomavirus type 16 E6 oncoprotein. Oncogene. 1998;16:1825‐1838. 9583680 10.1038/sj.onc.1201711

[18] Scott S , Travis D , Whitby L , Bainbridge J , Cross NC , Barnett D . Measurement of BCR‐ABL1 by RT‐qPCR in chronic myeloid leukaemia: findings from an International EQA Programme. Br J Haematol. 2017;177:414‐422. 28295199 10.1111/bjh.14557

[19] National Genetics Reference Laboratories . Armored RNA as reference material for standardisation of BCR‐ABL RQ‐PCR methods: report of field trial evaluation. NGRLW_aRNA_BCR_ABL_1.0. http://www.ngrl.org.uk/Wessex/downloads/Word/NGRLW_aRNA_BCR_ABL_1.0.d-oc. Accessed June 7, 2017.

[20] Brown JT , Laosinchai‐Wolf W , Hedges JB , et al. E‐stablishment of a standardized multiplex assay with the analytical performance required for quantitative measurement of BCR–ABL1 on the international reporting scale. Blood Cancer J. 2011;1:e13‐e21. 22829126 10.1038/bcj.2011.10 PMC3255280

[21] Zhan S , Li J , Xu R , Wang L , Zhang K , Zhang R . Armored long RNA controls or standards for branched DNA assay for detection of human immunodeficiency virus type 1. J Clin Microbiol. 2009;47:2571‐2576. 19494069 10.1128/JCM.00232-09 PMC2725685

[22] DuBois DB , Winkler MM , Pasloske BL . Ribonuclease resistant viral RNA standards: U.S. Patent 5,677, 124 p. 1997–10‐14.

[23] Pasloske BL , Walkerpeach CR , Obermoeller RD , Winkler M , DuBois DB . Armored RNA technology for production of ribonuclease‐resistant viral RNA controls and standards. J Clin Micro‐biol. 1998;36:3590‐3594. 10.1128/jcm.36.12.3590-3594.1998 PMC105245 9817878

[24] Gruden K , Cankar K , Blejec A . Calculation of measurement uncertainty in quantitative analysis of genetically modified organisms using intermediate precision a practical approach. J AOAC Int. 2007;90:582‐586. 17474528

[25] Liu Y‐f , Zhu Y‐m , Shen S‐h , et al. Molecular response in acute promyelocytic leukemia: a direct comparison of regular and real‐time RT‐PCR. Leukemia. 2006;20:1393‐1399. 16728984 10.1038/sj.leu.2404262

[26] Zhang T , Grenier S , Nwachukwu B , et al. Inter‐laboratory comparison of chronic myeloid leukemia minimal residual disease monitoring: summary and recommendations. J Mol Diagn. 2007;9:421‐430. 17690211 10.2353/jmoldx.2007.060134 PMC1975095

[27] ISO . Statistical Methods for Use in Proficiency Testing by Interlaboratory Comparisons M. Inter‐national organization for standardization, 2005.

[28] Barbaric D , Dalla‐Pozza L , Byrne JA . A reliable method for total RNA extraction from frozen human bone marrow samples taken at diagnosis of acute leukaemia. J Clin Pathol. 2002;55:865‐867. 12401828 10.1136/jcp.55.11.865 PMC1769789

[29] Branford S , Cross N , Hochhaus A , et al. Rationale.for the recommendations for harmonizing current methodology for detecting BCR‐ABL transcripts in patients with chronic myeloid leukaemia. Leukemia. 2006;20:1925‐1930. 16990771 10.1038/sj.leu.2404388

[30] Foroni L , Wilson G , Gerrard G , et al. Guidelines for the measurement of BCR‐ABL1 transcripts in chronic myeloid leukaemia. Br J Haematol. 2011;153:179‐190. 21382019 10.1111/j.1365-2141.2011.08603.x

