# Supplementary material for: External quality assessment for PML‐RARα detection in acute promyelocytic leukemia: Findings and summary
Source: J Clin Lab Anal. 2019 May 26;33(6):e22894. doi: 10.1002/jcla.22894 (PMC6642306; doi:10.1002/jcla.22894)
Supplement: Supplementary file 1 [file JCLA-33-e22894-s001.docx]

**Supplemental Material 1.** APL simulated clinical case

The patient was 41 year old male. Due to Anemia, bleeding gums in admission examination, no history of blood disease, no history of tumor radiotherapy and chemotherapy; Physical examination showed sternal pain, skin and mucous membranes extensive bleeding, petechiae, heart, liver and kidney function was­­­­­­­­­ acceptable. Blood counts at presentation were WBC 3,000/mm^3^, hemoglobin 8.0g/dl, and platelets 65,000/mm^3^; D-dimer: 25μmol /L, LDH: 428U/L. The bone marrow cellularity showed significant proliferation of nucleated cells, myeloblasts 0.020, promyelocytes 0.185, myelocytes 0.060, non-erythroid nucleated cell count (NEC) ratio> 60%, faggot Auer body observed. Immunophenotyping by flow cytometry revealed that CD13 +, CD33 +, HLA-DR-, CD34-.
