# Supplementary material for: External quality assessment for PML‐RARα detection in acute promyelocytic leukemia: Findings and summary
Source: J Clin Lab Anal. 2019 May 26;33(6):e22894. doi: 10.1002/jcla.22894 (PMC6642306; doi:10.1002/jcla.22894)
Supplement: Supplementary file 2 [file JCLA-33-e22894-s002.docx]

**Supplemental Material 2.** The quantitative validation results of the *PML-RARα* FG EQA panel

| EQA panel | Sample No. | One-step method | | Two-step method | |
| --- | --- | --- | --- | --- | --- |
|  |  | FG/CG ratio | MRD value | FG/CG ratio | MRD value |
| A | A1711 | 10.41% | 1 | 12.23% | 1 |
|  | A1712 | 2.77% | 0.2660 | 2.66% | 0.2175 |
|  | A1713 | 0 | Negative | 0 | Negative |
|  | A1714 | 0 | Negative | 0 | Negative |
|  | A1715 | 0.016% | 0.0015 | 0.014% | 0.0011 |
| B | B1721 | 10.01% | 1 | 9.23% | 1 |
|  | B1722 | 1.49% | 0.1489 | 1.74% | 0.1885 |
|  | B1723 | 0 | Negative | 0 | Negative |
|  | B1724 | 0 | Negative | 0 | Negative |
|  | B1725 | 0.02% | 0.0020 | 0.011% | 0.0012 |
| C | C1731 | 128.81% | 1 | 124.59% | 1 |
|  | C1732 | 0 | Negative | 0 | Negative |
|  | C1733 | 0.02% | 0.00016 | 0.028% | 0.00022 |
|  | C1733 | 0 | Negative | 0 | Negative |
|  | C1735 | 0.20% | 0.00155 | 0.19% | 0.00152 |
|  | C1736 | 7.94% | 0.0616 | 9.76% | 0.0783 |
